# Transcriptional Regulation of *YWHAZ*, the Gene Encoding 14-3-3ζ

**DOI:** 10.1371/journal.pone.0093480

**Published:** 2014-04-01

**Authors:** Andrea Kasinski, Xueyuan Dong, Fadlo R. Khuri, Jeremy Boss, Haian Fu

**Affiliations:** 1 Program in Genetics and Molecular Biology, Emory University, Atlanta, Georgia, United States of America; 2 Department of Pharmacology, Emory University, Atlanta, Georgia, United States of America; 3 Department of Microbiology and Immunology, Emory University, Atlanta, Georgia, United States of America; 4 Department of Hematology and Medical Oncology, Emory University, Atlanta, Georgia, United States of America; North Carolina State University, United States of America

## Abstract

Aberrant expression of oncogenic 14-3-3 proteins is correlated with poor survival of cancer patients. While the underlying mechanism of the abnormal expression in tumors remains elusive for the six oncogenic 14-3-3 isoforms; the potential involvement of a transcriptional component has been suggested. Unfortunately, little experimental data has been reported to support this hypothesis. In this study we describe the genetic structure of *YWHAZ*, the gene encoding 14-3-3ζ, including the identification of previously unreported transcript variants. In total, five transcript variants were revealed and their expressions confirmed in a panel of cell lines. Expressed sequence tag (EST) database mining and *in vitro* rapid-amplification of cDNA ends (RACE) confirmed that one variant, 1c, represents >80% of the expressed transcripts, which is also the most efficiently translated. An analysis of the proximal promoter of this variant revealed a functional Cyclic-AMP Response Element (CRE). Factors that bound to the CRE element were recognized through fractionation and subsequent EMSAs. This analysis identified CREB and ATF-1 as the trans-interacting factors. Cell-based assays confirm that ATF-1, and to a lesser extent CREB, bind the endogenous *YWHAZ* promoter especially under TNF-α stimulating conditions. In support of a role of ATF-1 in the regulation of *YWHAZ*, silencing of ATF-1 resulted in a marked reduction in two of the five *YWHAZ* transcripts. These data suggest a novel mechanism for the transcriptional regulation of a major pro-survival gene, *YWHAZ*, by ATF-1.

## Introduction

The 14-3-3 proteins are a family of acidic, dimeric, conserved proteins found in all eukaryotes examined. There are seven isoforms (β, γ, ε, η, σ, τ and ζ) encoded by seven distinct genes in mammals, which act as master regulators in the cell. The pathways that they regulate converge on various aspects of cell survival signaling through their coordinated modulation of cell cycle progression, proliferation, transformation, and the onset of differentiation and senescence [1]. The ability to control such events depends on binding of a 14-3-3 dimer to over 200 known client proteins at phosphoserine/phosphothreonine-containing motifs [2]. The majority of 14-3-3 ligands contain one of three well-defined binding motifs (RSXpS/TXP, RXFXpS/TXP, or pS/T(X_1–12_)-COOH, where pS/T, F and X denote a phosphorylated serine or threonine residue, an aromatic amino acid and any amino acid, respectively) [3]–[6]. However, ligands have been identified that can bind 14-3-3 in a phosphorylation-independent manner as well. Regardless, these binding events lead to altered client protein sub-cellular localization (eg. BAD, CDC25), modification of enzymatic function (eg. RAF-1, serotonin-N-acetyl transferase, CHK1, and WEE1), and/or the formation of dynamic multi-protein complexes (eg. RAF-BCR and RAF-A20) [7]–[14].

The majority of the work in the field has focused on how 14-3-3 proteins regulate the function and sub-cellular localization of other proteins with limited attention given to how 14-3-3 proteins themselves are regulated. However, it has been reported that many 14-3-3 family members, including 14-3-3β and ζ, can undergo post-translational modification by phosphorylation [15], [16]. One such modification at this level disrupts 14-3-3 dimer formation which is necessary for subsequent client protein interaction [15]. Indeed, 14-3-3 proteins function as hetero- and homodimers with particular heterodimeric pairs having specificity and/or affinity for individual client proteins. As such, the level of each individual isoform in the cell and its localization can dictate successive events. It is expected that alterations of 14-3-3 proteins due to mutation or expression of specific isoforms can result in global changes in the cell.

The ability of 14-3-3 dimers to bind to and regulate oncogenic proteins and tumor suppressors such as RAF-1 and BAD, respectively, point to a role for 14-3-3 proteins in cancer. Of the seven isoforms, six “canonical” members are referred to as oncogenic. The only isoform with tumor suppressive properties is 14-3-3σ, which is silenced in the epithelial cells in a sub-set of breast cancers leading to decreased genomic instability [17]. As one of the only isoforms intensely studied at the genomic level, it was determined that gene silencing in these tumors occurs at an epigenetic level due to methylation of a CpG island within the promoter of *SFN*, the gene encoding the 14-3-3σ protein. Additionally, a functional binding site for the tumor-suppressor p53 is present in the promoter of *SFN* [18]. In response to DNA damage, p53 is recruited to the promoter resulting in gene activation leading to G2-M arrest.

Although the transcriptional contribution of the remaining six isoforms is less studied, recent reports suggest a genetic component to their regulation. Various groups have shown that gene amplification and increased transcription of the oncogenic isoforms are often present in cancerous tissue and may contribute to protein elevations. For example, Qi and colleagues provide evidence for increased transcript levels of multiple 14-3-3 isoforms in lung cancer, while Neal and colleagues determined that gene amplification might be one mechanism leading to elevated 14-3-3ζ levels in breast cancer [19], [20]. The *YWHAZ* promoter has also been shown to be under the control a functional androgen receptor binding site that, in the presence of androgen, induces 14-3-3ζ expression in prostate cancer cell lines [21]. These studies suggest a clear role for promoter-driven activation of *YWHAZ* and perhaps involvement of the 5′UTRs since they are also quite divergent, advocating for the possibility that individual family members may have additional control mechanisms in place at both the transcriptional and translational level. Moreover, as identified in the database, multiple transcript variants exist for both 14-3-3β and ζ that may add another layer of complexity to the regulation of these two isoforms [22].

In this report we focus on the genetic organization and regulation of *YWHAZ*, the gene encoding 14-3-3ζ. We identified a total of 5 transcript variants, each of which contains a different 5′UTR. We show that the UTR's are unique in their ability to translate the protein. Moreover we identify that of the five variants, one makes up the bulk of the *YWHAZ* mRNA. We present evidence that this particular variant is transcriptionally expressed at levels higher than the other four variants and is also the most readily translated. We further show that this variant is regulated by a CRE element found in the proximal promoter of the gene and confirm that ATF-1 and CREB bind to the putative CRE element *in vitro* and that ATF-1 binds the endogenous promoter. Knockdown of ATF-1 diminishes two of the five transcript variants in a dose-dependent manner. This suggests a novel mechanism for 14-3-3ζ regulation. Our report represents the first to examine transcriptional mechanisms that control any of the oncogenic 14-3-3 family members.

## Results

### *YWHAZ* is expressed in the form of at least five different transcript variants

**Figure 1:**
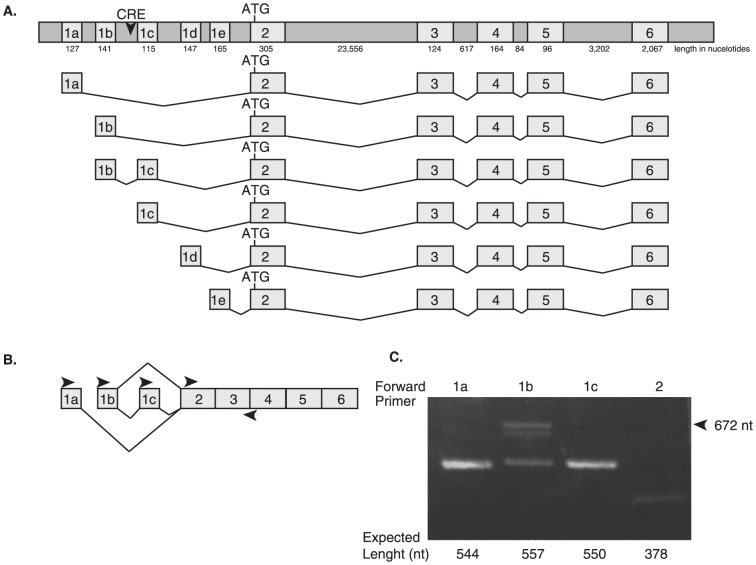
Transcript variants of *YWHAZ*. (**A**) Genomic locus for *YWHAZ*. Five different first exons are indicated as 1a, 1b, 1c, 1d and 1e. ATG represents the start methionine. The putative CRE element is depicted as a downward arrowhead within the intron preceding exon 1c. The number of nucleotides contained within the introns and exons is labeled below each respective feature. The six *YWHAZ* transcript splice variants are shown below. (**B**) Representation of primer pair locations used for reverse transcription PCR to identify variants. Right arrowheads indicate forward primers used in this study, while a left arrowhead spanning the boundary of exon 3 and 4 depicts the one universal reverse primer. (**C**) Transcript variants 1a, 1b and 1c and total *YWHAZ* transcript levels (forward primer in exon 2) were identified in mRNA pools from HeLa cells using the primers indicated. A higher molecular weight product (672 nt) was amplified with the forward primer for exon 1b (arrowhead). All products were gel purified, sequenced, and confirmed to be variants of *YWHAZ*. The higher molecular weight product for 1b (lane 2) was identified as containing exon 1b spliced to exon 1c followed by exon 2.

The gene that encodes for 14-3-3ζ, *YWHAZ*, is located on chromosome 8 spanning just over 35 kilobases. There are six reported exons for this gene in the database [22], the first lying entirely within the 5′ untranslated region (UTR) (Figure 1A). At the start of this work, two transcript variants were predicted for this gene (NM_003406.2, and NM_145690.1) each with an independent exon 1 (1a and 1c) [23]. As the study unfolded several additional variants were reported in the RefSeq database [23]; however, their contribution to 14-3-3ζ expression remains unknown. To systematically discover and examine the structure and functions of *YWHAZ* variants, we preformed RNA-ligase-mediated (RLM) RACE. This technique aids in the identification of the 5′ transcriptional start sites and helps to indicate the relative abundance of individual transcripts in an mRNA pool. Using this method, we confirmed the expression of the two originally reported variants (beginning with exon 1a and exon 1c) and identified two additional splice variants (one beginning with exon 1b and the other beginning with exon 1e) (Figure 1A). An additional variant was later discovered as a splice variant containing exon 1b spliced directly to exon 1c. This variant was found as a higher molecular weight product following reverse transcription (RT) PCR (Figure 1C). Subsequent purification and sequencing identified it as the fifth *YWHAZ* transcriptional variant. The final variant depicted in Figure 1A is suggested to begin with exon 1d (NM_001135701) based on deposited expressed sequence tags. We were unable to confirm expression of this variant in the ten cell lines we tested; however the possibility exists that its expression may be induced in different cells or tissues in a context specific manner.

**Figure 2:**
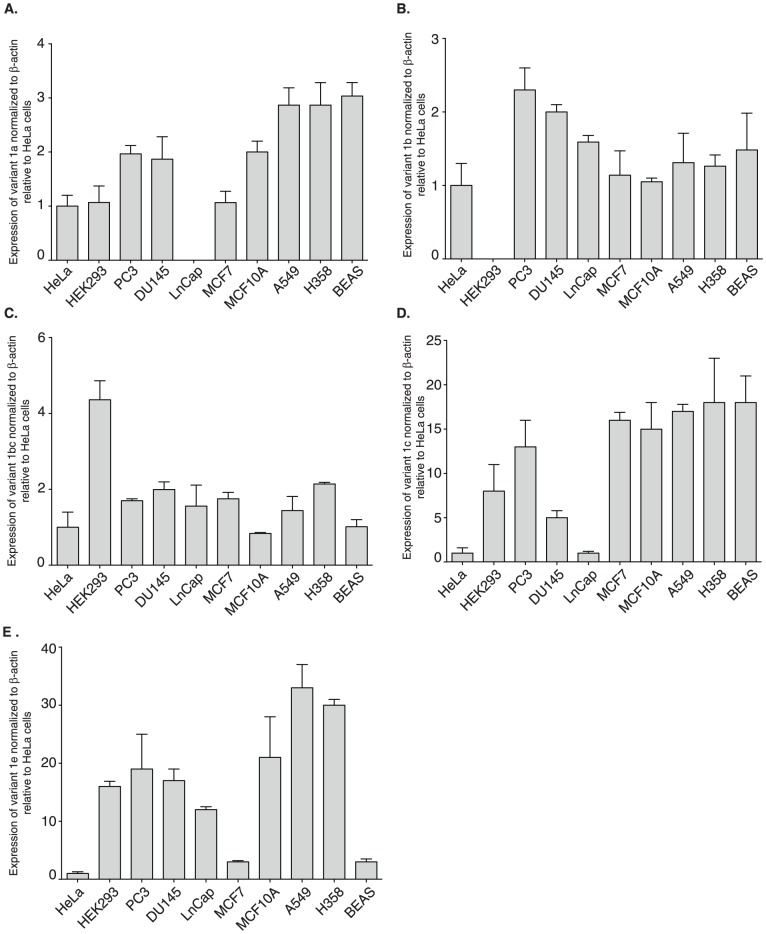
Transcript variants are differentially expressed. cDNA was used in qRT-PCR reactions to determine the relative expression of each of the five transcript variants (**A**) variant 1a (**B**) variant 1b (**C**) variant 1bc (**D**) variant 1c (**E**) variant 1e. Data shown are normalized to β-actin and graphed relative to normalized levels obtained from HeLa cell RNA. Error bars shown represent the standard deviation obtained from three technical represents. Two additional biological represents show similar trends. Colorectal cell lines: HeLa; prostate cancer cell lines: PC3, DU145, LnCap; breast cancer cell lines: MCF7, MCF10A, lung cancer cell lines: A549, H358; normal lung cell line: BEAS.

To confirm the expression of the five variants that we identified from our RACE data, quantitative real-time RT-PCR (qRT-PCR) analysis was performed (Figure 2). We observed changes in expression between cell lines and between variants. Interestingly, we show a positive correlation between expression of four variants (1a, 1b, 1c and 1e) and the aggressiveness of the prostate cancer cell lines. In all cases the least aggressive of the prostate cancer cell lines, LNCap, had the lowest level of expression of all four variants while the highly metastatic PC3 cells have highest levels of expression for variant 1b and 1c (For variant 1c: *p*-value = 0.01 for PC3 cells relative to DU145, *p*-value = 0.002 for PC3 cells relative to LNCap, *p*-value = 0.001 for DU145 relative to LNCap).

### *YWHAZ* variant 1c is the most efficiently transcribed and variants containing exon 1c are the most highly translated

While multiple transcripts of 14-3-3ζ have been confirmed through this work, the contribution of each transcript variant to the overall expression of 14-3-3ζ remains unknown. We therefore examined two mechanisms important for regulating cellular protein levels. The first assessed the relative abundance of the different transcript variants while the second evaluated each of the 5′-UTR's for their ability to direct translation.

**Figure 3:**
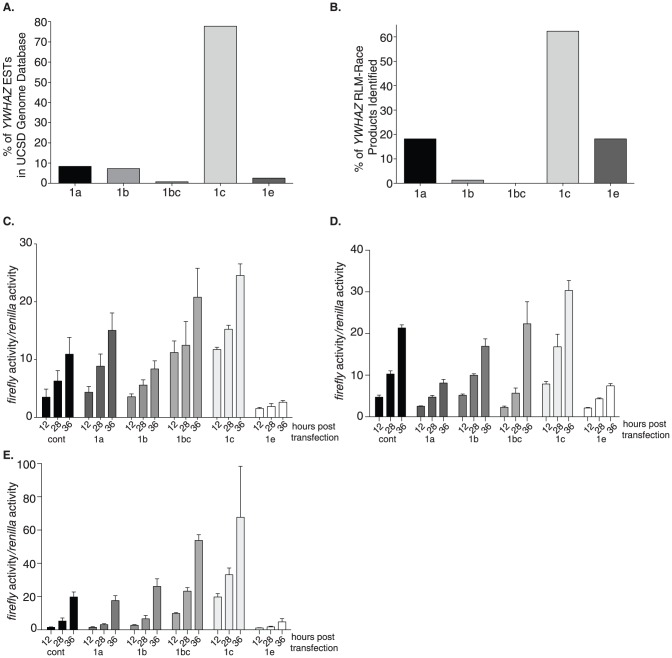
The 5′-UTR of *YWHAZ* variant 1c is the most highly transcribed and efficiently translated. (**A**) A total of 265 *YWHAZ* transcripts derived from various human cell lines and tissues were deposited in the UCSD Genome Database at the time of this study. These are subdivided into those belonging to the five different transcript variants and are graphed as percentages relative to the total. (**B**) RLM-RACE clones (77) obtained from MCF7 and HeLa cells were sequenced, pooled, and grouped according to the exon that they began with. They are represented as the percent of clones identified that contained that particular variant. (**C**) HeLa (**D**) H1299 and (**E**) HEK-293 cells were transfected with luciferase reporter vectors containing each of the 5′-UTR's and *renialla* control plasmid to normalize luciferase activity. Cells were lysed at 12, 28 and 36 hours post transfection. Isolated protein was assayed for luciferase activity. Data are graphed as *firefly* activity relative to *renilla* activity and are normalized to levels at 12 hours. Data are shown from one of three independent biological replicates. Error bars for C, D, and E represent standard deviations obtained from four technical replicates.

It is likely that one or more of these transcripts may be playing a larger role than the others based on the abundance of message produced. To determine if any of the transcripts were more highly expressed than others, we first looked *in-silico* at the human expressed sequences tags (EST) deposited in the UCSC Genome Database [22]. Although all five variants were identified, of the 265 EST's in the repository, greater than 75% began with exon 1c (Figure 3A). The variants beginning with 1a or 1b were the next highest representing fewer than 20% combined. The other variants, although present, were only a minor fraction of the total. To confirm this finding in cultured cells, we evaluated our 5′-RLM-RACE data, which allowed us to determine the relative abundance of the transcript variants in our mRNA pool. In this small screen of 77 clones our experimental findings supported the EST database results; the majority of the clones pulled out of the screen began with variant 1c (Figure 3B). We were able to identify all variants except for the splice variant containing exons 1b and 1c, the variant least represented in the EST database as well (1/265). There is a slight elevation in the amount of variant 1e represented in our RLM-RACE screen with a similar decrease seen for variant 1b when compared to the EST database search. Importantly, variant 1c was found to be the most highly represented transcript *in silico* and in our *in vitro* assays.

Although elevated transcript levels often correlate with increased protein abundance, this is not always the case. Therefore we evaluated the level of control contained within the unique 5′-UTR of these variants, which can dictate translation efficacy. Each of the transcript variants encodes for the same protein. The start methionine resides in exon 2 and all the variants identified in this study share exons 2 through 6 (Figure 1A). The inconsistencies between the transcripts reside solely in exon 1, which encompasses the majority of the 5′-UTR. The ability of a protein to be translated efficiently depends heavily on the sequence within the 5′-UTR of the message. To assay the level of translation elicited by each of the 5′-UTR's, the five UTR's were cloned upstream of a luciferase reporter vector fusing the ATG of *YWHAZ* with the ATG for the luciferase gene. Transcript levels of *luciferase* and *renilla* were monitored in Hela cells at 12, 28, and 36 hours to confirm equal expression (Figure S1). All vectors expressed near equal levels of *luciferase* transcript levels relative to *renilla*, suggesting that changes we observed in luciferase activity could be attributed to alteration in translation and were not because of inconsistencies in message levels. At each of the time-points, the relative level of luciferase activity was determined in Hela, H1299, and Hek293 cells. In all three cell lines the 1c UTR and 1b/c slice variant UTR generated the most luciferase activity (Figure 3C–E). The 1a and 1b UTR's were relatively equal in their translation efficiency, while the 1e UTR generated luciferase activity lower than the empty vector control and therefore may have repressible elements contained within the sequence. These data suggests that the transcript-specific UTR's function independently to control protein translation. Further, the 5′-UTR variants that contain exon 1c, variant 1c and 1b/c, support the strongest level of translation suggesting that a *cis*-regulator element in the region might be responsible for the enhanced translation.

### Identification of a CRE regulatory region in the proximal promoter of variant 1c

**Figure 4:**
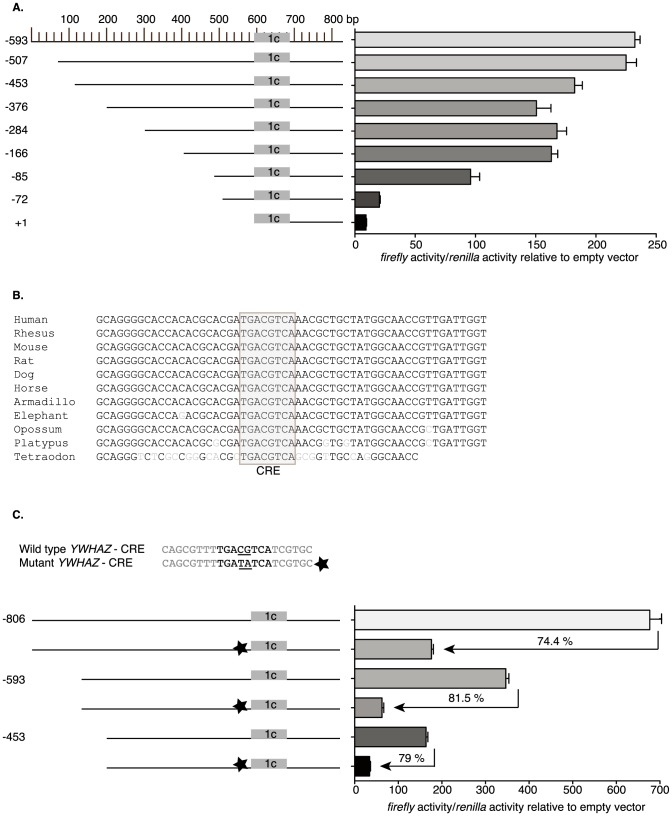
Functional CRE element in *YWHAZ* transcript variant 1c. (**A**) The proximal promoter for transcript variant 1c was cloned upstream of a luciferase reporter vector. Truncations of the promoter region were generated and cotransfected with *renilla* control vector and assayed for relative luciferase activity 24 hours later. Data are graphed as *firefly* relative to *renilla* over empty vector control and represent averages of three technical experiments +/− one standard deviation. (**B**) The sequence between and surrounding the *YWHAZ* truncations (−72 and −85) was aligned from human to *Tetraodon*. Light grey nucleotides indicate loss of conservation. The grey box containing 100% sequence conservation contains the putative CRE element. (**C**) Schematic of the sequence surrounding the CRE element and the two-nucleotide mutation generated (star) is shown. HeLa cells were transfected with one of three variant 1c constructs with or without CRE mutations engineered into them and a *renilla* control reporter plasmid. Data are graphed as *firefly* relative to *renilla* over empty vector control and represent the average of three independent experiments +/− one standard deviation. Loss of activity for each of the three mutated reporters is indicated.

Exon 1c supports the greatest translation efficiency (Figure 3C–E). Further, the transcript level of variant 1c is the most highly expressed as determined from both the EST database and from our experimental data (Figure 3A and 3B). We therefore began to identify *cis*-regulatory regions necessary for expression from the promoter of variant 1c. The first 593 base pairs of the proximal promoter were placed upstream a luciferase reporter system and deletions of the promoter region were generated. When the truncated reporters were evaluated in reporter assays, two regions contained putative *cis*-activating regulatory sequences (Figure 4A). A decrease of approximately 48% in luciferase reporter activity was observed between the −166 and −85 constructs with about a 79% decrease seen between −85 and −72.

In an effort to determine if any regions within these sequences were evolutionarily conserved, we performed comparative genomics on both regions. Although a high degree of nucleotide similarity exists, we were able to determine a single region between the −85 and −72 constructs that was fully conserved down to *Tetraodon nigroviridis* that contained a conserved transcription factor binding site (Figure 4B). The conserved region of an 8 bp palindromic sequence is most similar to a cyclic-AMP-response-element (CRE) (refer to Figure 1A for location within *YWHAZ*). Indeed, functional CRE elements are usually found within approximately 200 bps of the transcriptional start-site [24], which holds true for variant 1c.

### A putative CRE element is necessary for basal activation from the *YWHAZ* promoter

CRE elements are characteristically 8-bp palindromic sequence (TGACGTCA); however, a truncated, albeit less active, half palindrome is also functional [25]. With reference to the 8-bp palindrome, an unmethylated CG dinucleotid is necessary for the expression of CRE target genes [26]. In order to determine if the CRE element that was identified in this study was functional, the critical GC dinucleotide within the palindrome was mutated to TA in three independent reporter vectors (Figure 4C), which included a reporter containing an additional 213 nucleotides further upstream (−806) than those used in our proximal promoter analysis. Mutation of these two key residues within the promoter sequence of all three reporters reduced activity by greater than 70%, suggesting that the CRE element is functional in our cell culture assay (Figure 4C). The mutated reporter containing a larger portion of the 5′-UTR (−806) retained more activity than the other smaller reporters (−593, and −453): 25.6% activity retained relative to 18.5% and 21% respectively. This suggests that additional *cis*-regulatory elements within the 213 nucleotides of the −806 construct may be involved in the 5% increase in activity.

### Nuclear proteins bind the CRE element of *YWHAZ*

**Figure 5:**
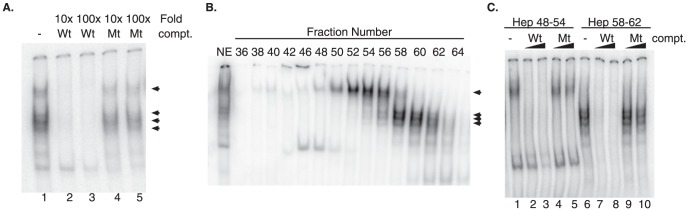
Specific binding of nuclear proteins to the putative CRE element *in vitro*. (**A**) EMSAs were performed using crude nuclear extracts prepared from HeLa cells incubated with a 28-nt-P^32^ labeled probe surrounding the putative CRE element and were resolved on nondenaturing PAGE (lane 1). Ten or one-hundred fold excess of either wild-type (lanes 2 and 3) or mutant (lanes 4 and 5) competitor oligo was added. (**B**) Heparin eluted fractions were assayed for their binding ability by EMSA. Multiple complexes were identified (arrowheads) that could be separated into two pools with peak EMSA activity. (**C**) Fractions with positive binding activity were pooled into two separate groups: fractions 48–54 and fractions 58–62. Each of the pooled fractions was subject to competition assays with either 10 or 100 fold excess of wild-type or mutant competitor.

Our luciferase experiments suggest that the putative CRE element was responsible for basal promoter activity; therefore, an electrophoretic mobility shift assay (EMSA) was used to identify if any nuclear factors could bind the sequence *in vitro*. A 28-nucleotide probe containing sequences flanking the *YWHAZ*-CRE region was generated, labeled with ^32^P, and assayed for its ability to shift in the presence of crude nuclear extract obtained from HeLa cells. Nondenaturing polyacrylamide gel electrophoresis of the bound probe revealed at least 4 independent CRE specific complexes (Figure 5A). All four complexes were confirmed to be specific as they could be competed away with an excess of unlabeled probe (Figure 5A, compare lane 1 to lane 2 and 3). The specificity of this interaction was further evaluated by including an unlabeled competitor engineered with a mutation within the CRE element. This mutant sequence could not compete with the radioactively labeled wild-type probe for binding, suggesting that the complexes seen in our EMSA are dependent on an intact CRE element (Figure 5A, lanes 4 and 5).

To separate and concentrate the different nuclear factors for further analysis, 20 mg of purified nuclear proteins were bound to a heparin agarose affinity column. Bound proteins were eluted and collected over a salt gradient and every-other fraction was subjected to EMSA analysis to identify fractions containing CRE binding activity. As evident in Figure 5B, the fractionation technique separated the shifted complexes into two major populations: one group in fraction numbers 48–54 and a second in fractions 58–62. These fractions were independently pooled and used to further characterize the proteins that bound the *YWHAZ-*CRE probe. Competition assays confirmed that both pooled populations contained complexes that bound to the wild-type CRE element. This was specific as inclusion of excess mutant sequence was unable to disrupt complex formation while excess unlabeled wild-type sequence was able to (Figure 5C).

### ATF-1 and CREB bind to the CRE element of *YWHAZ*

**Figure 6:**
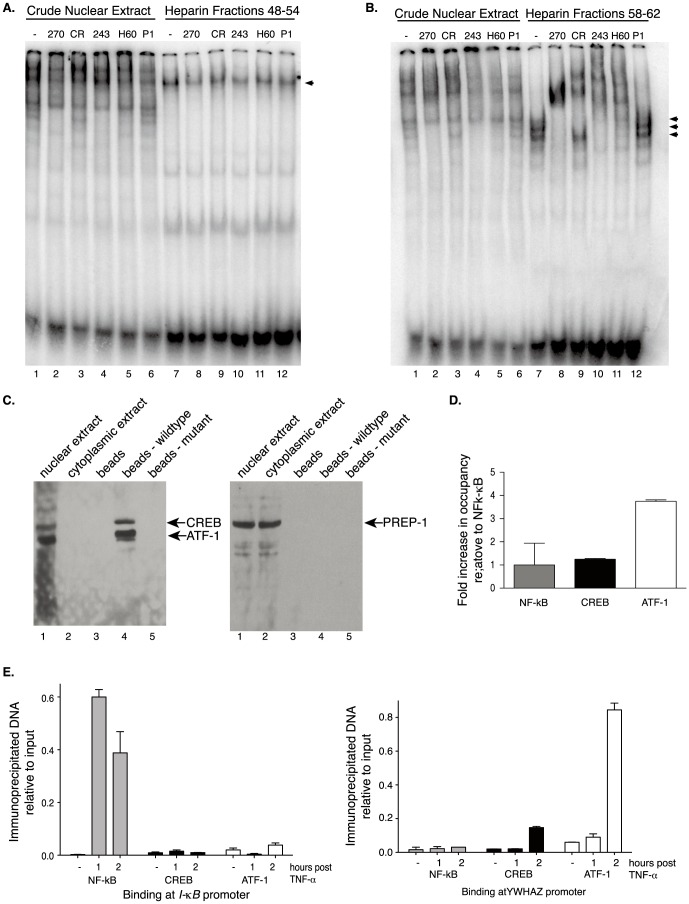
CREB and CREB family members bind to the *YWHAZ* promoter. EMSAs were performed as in Figure 5. Antisera in the reaction include: 270 (CREB, CREM and ATF-1), CR (CREB); 243 (ATF-1), H60 (ATF-1), P1 (PREP-1; negative control). Supershift assay of crude nuclear extract and fractions 48–54 (**A**) or 58–62 (**B**). Arrowheads represent the major complexes identified after heparin fractionation. (**C**) Nuclear extracts were incubated with streptavidin beads conjugated to wild-type or mutant biotinylated oligos. Bound proteins were resolved by SDS-PAGE, transferred to nitrocellulose membrane, and probed with antisera against CREB, ATF-1, or PREP-1. (**D–E**) HeLa cells were cultured in the absence (D) or presence (E) of TNF-α followed by ChIP. ChIP assays were performed using antisera against ATF-1, CREB or NF-κB (p65). Co-immunoprecipitated DNA was amplified with primer sets to *YWHAZ* and I-κB promoters and is represented as amplified product relative to product obtained from 1/30 the amount of input, except in D where the data are further normalized to NF-κB occupancy. Standard deviations are indicated from three technical replicates. Two additional biological replicates show similar trends.

CREB and CREB family members, activating transcription factors (ATF)1–7 and CREM, are basic leucine zipper domain (bZIP) transcription factors that bind CRE elements as both hetero- and homo-dimers of each other acting to induce or repress the expression of target genes. Our luciferase experiments identified a putative CRE activating element within the proximal promoter of *YWHAZ.* Likewise a probe encompassing this element shifted in our EMSA; therefore, it seemed likely that CREB or one of the CREB family members was binding to this element. To test this hypothesis, supershift experiments were performed on the enriched heparin pooled fractions. No apparent shift was observed when antisera against CREB or CREB family members was included with pooled fractions 48–54 (Figure 6A, lanes 8–11). However, both CREB and ATF-1 antisera shifted all three bands in fractions 58–62 (Figure 6B). Lane 2 (crude extract) and 8 (heparin fractions 58–62) showed shifts of all three major bands when a single antisera recognizing CREB, CREM and ATF-1 was included. Supershifts with a CREB specific antibody confirmed the presence of CREB in two of the three complexes (Figure 6B, lanes 3 and 9). ATF-1 was identified as a component in all of the major complexes (lanes 4, 5, 10 and 11). Importantly, an antibody to PREP-1, an unrelated transcription factor, was unable to produce a shift in any of the bands from either pooled fraction (Figures 6A and 6B, lanes 6 and 12). This suggests that an ATF-1/CREB heterodimer may be acting in one complex while an ATF-1 homodimer or an ATF-1 heterodimer with a yet to be identified additional factor may be contained in a separate complex. The identification of the CREB/ATF-1 independent factors bound in fractions 48–54 has yet to be determined.

To further confirm the binding of CREB and ATF-1 to the CRE element, a streptavidin agarose pulldown assay (SAPD) was performed. Double stranded biotinylated *YWHAZ* oligos that spanned the CRE element were generated and attached to streptavidin beads. After incubating the oligo-bound beads with nuclear extract, beads were collected, washed, and precipitated proteins were resolved by SDS-PAGE. A single antibody that recognizes both CREB and ATF-1 was used to detect the abundance of these two proteins in the *YWHAZ* oligo-protein complex. As expected Figure 6C lane 1 shows that both CREB and ATF-1 present in the nuclear fraction and absent from the cytoplasmic extract. Moreover, both proteins were enriched following incubation with wild-type-bound beads (Figure 6C, compare lane 4 to lane 5). The beads bound to the mutant sequence failed to pull down either of these proteins, confirming specificity for the intact CRE element. Additionally, binding was specific for CREB and ATF-1; PREP-1, a non-specific factor, failed to come down with either of the bead preps. This data supports the EMSA findings, that CREB and ATF-1 can bind the wild-type CRE element of *YWHAZ in vitro*.

To determine if either of these factors could occupy the CRE element in cells chromatin immunoprecipitation (ChIP) was used. Interestingly, only antisera against ATF-1 immunoprecipitated *YWHAZ*-CRE promoter DNA under the basal conditions tested (Figure 6D). There was approximately a four-fold increase in ATF-1 occupancy at the *YWHAZ* promoter relative to the p65 subunit of NF-κB or CREB.

### ATF-1 is recruited to the endogenous *YWHAZ* promoter after stimulation with TNF-α

Previous reports suggest that both CREB and ATF-1 can be recruited to promoters after stimulation with TNF-α [27]. Based on the ability of TNF-α to activate an apoptotic response, it seemed likely that one mechanism by which cancer cells may act to evade TNF-α induced apoptosis would be to counter balance this response by inducing pro-survival factors such as 14-3-3ζ. Indeed this is the case in many cancer cells that are subject to pro-apoptotic stimuli where survival pathways, such as the nuclear-factor kappa B (NF-κB), are activated in response to TNF-α [28]. To determine if the *YWHAZ* promoter acts in a similar fashion with respect to CREB and ATF-1, we performed the ChIP experiment after exposure to TNF-α. HeLa cells were cultured with TNF-α (10 ng/ml) for 0, 1 or 2 hours, followed by ChIP with CREB, ATF-1 or the p65 subunit of NF-κB. As expected, TNF-α induced binding of NF-κB at the κB site in the promoter of the inhibitor of kappa B (I-κB), while neither CREB nor ATF were detected at the I-κB promoter (Figure 6E, left panel). On the contrary, antisera against ATF-1, and CREB to a lesser extent, immunoprecipitated a significant amount of *YWHAZ* promoter DNA after stimulation (Figure 6E, right panel). These results are consistent with earlier reports that TNF-α triggers modifications of ATF-1 and CREB and their subsequent binding to CRE elements [27].

We attempted to show that TNF-α induces expression of the *YWHAZ* transcript variant 1c and activates the luciferase reporters. In both cases TNF-α was incapable of inducing expression. It is likely that additional signaling events or cofactors are required after binding of ATF-1 to the promoter for inducing this enhanced expression. However, regardless of downstream events that are required for expression, we show that ATF-1 is indispensable for full expression. Suggesting that ATF-1, while perhaps not sufficient, is required.

### ATF-1 regulates expression of *YWHAZ* transcript variants 1c and 1e

**Figure 7:**
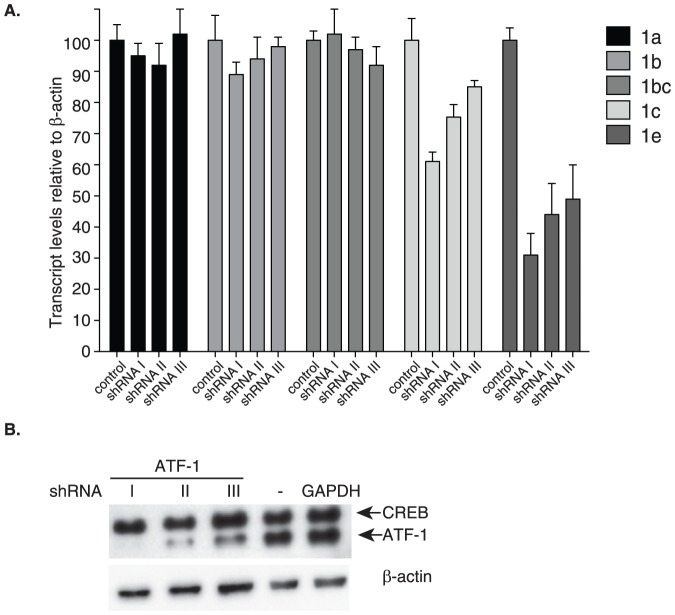
ATF-1 regulated expression of transcript variants 1c and 1e. HeLa cells were transfected with three different shRNAs targeted to the 3′ UTR of ATF-1 (I, II, and III). Four days following transfection, total RNA was isolated and reverse transcribed. Amplification of *YWHAZ* transcripts was carried out with transcript specific primers by semi-quantitative PCR followed by densitometry analysis. Data are normalized to β-actin transcript levels and graphed relative to RNA evaluated from control shRNA transfected cells. (**B**) Western showing knockdown of ATF-1 four days following transfection. Antibodies for CREB and ATF-1 were multiplexd. β-actin serves as a loading control. Error bars shown represent the standard deviation obtained from three technical represents. Two additional biological represents show similar trends.

Mutations of the CRE element decreased activity of the *YWHAZ* promoter in reporter assays (Figure 4C). Since ATF-1 can bind this element, we next determined the requirement of ATF-1 in controlling endogenous *YWHAZ* transcription in cells with a RNA interference approach. A short haripin RNA (shRNA) targeting ATF-1 was expressed in HeLa cells. To circumvent the possibility of non-transfected cells confounding our data analysis, cells were placed under selection for 3 days prior to protein or mRNA extraction. Because the vector used in this study produced a polycistronic transcript containing both the shRNA and a sequence encoding green fluorescent protein (GFP), we were able to monitor GFP fluorescence as a proxy for shRNA expression. Three days after the cells were put under selection, greater than 85% expressed detectable levels of GFP (data not shown), indicating that shRNA expression in these cells was achieved. Time points beyond three days resulted in complete cell death due to ATF-1 knockdown, as control transfected cells still continued to proliferate. Therefore, the remaining experiments were conducted four days after shRNA transfection (three days following selection). The relative levels of the five transcript variants were determined by semi-quantitative PCR. Expression levels for transcript variants 1c and 1e were reduced in cells overexpressing shRNA to ATF-1 in a dose-dependent manner, while no apparent change was observed in the transcript levels for variants 1a, 1b, and 1b/c (Figure 7). This suggests that ATF-1 contributes to the expression of *YWHAZ* through controlling the transcription of two variants, one of which, variant 1c, is the most highly expressed and the most readily translated of the five variants.

## Discussion

Elevated expression of 14-3-3 isoforms have been correlated with poor outcome of cancer patients [29]–[31]. As such, targeting these pro-survival master regulators for therapeutic development is an intense focus of the field. Although many of the current efforts are aimed at inhibiting 14-3-3 client protein interactions, strategies to block the expression or function of a specific isoform remain to be established. Because all seven family members share a remarkable degree of amino acid conservation, targeting one isoform is challenging at this level. Additionally, knockout studies in yeast suggest that a certain amount of functional 14-3-3 protein is necessary for normal cellular growth and proliferation [32]. Therefore, targeting a specific isoform individually is expected to have added advantages. This is supported by recent findings that suggest dominant roles of a particular 14-3-3 isoform. For example, knockdown of 14-3-3γ alone results in BAX and BAD induced apoptosis while the contribution of 14-3-3ζ to anchorage independent growth was recently determined [33], [34]. This emphasizes the importance of isoform-associated cellular functions and the inability of the remaining isoforms to compensate for each other. As such there is a pressing need to better understand the regulation of the individual isoforms, which may offer an opportunity for therapeutic development through isoform specific inhibitors.

In this current study we show the genomic contribution to 14-3-3ζ expression. We present here for the first time the transcriptional regulation of this pro-survival 14-3-3 isoform. Five different transcript variants have been confirmed through this study, three of which have not yet been reported in any peer-reviewed publication. Furthermore, one of the transcripts, variant 1c, is the most dominant in its message levels and its translation efficiency. Finally, we show that the CREB family of transcription factors controls this major transcript variant.

The CREB family of transcription factors binds to CRE elements in the promoter and enhancers of multiple genes. In fact, more than 6,300 CRE elements have been identified in the human genome [35]; however, their functionality has not yet been determined. In our study we show that the CRE element within the proximal promoter of *YWHAZ* is indeed functional, as mutations in the element result in a marked reduction in reporter activity (Figure 4C). Indeed the expression of CREB and ATF-1 change during the progression of human tumors. CREB protein levels positively correlate with the metastatic potential of melanoma cells [36]–[40] and although ATF-1 is not identified in normal human melanocytes, it is easily identifiable in metastatic melanoma [36]–[40]. Furthermore, both CREB and ATF-1 were found to be overexpressed in hypoxic lung tissue [41]. This later study suggests that dysregulation of CREB and ATF-1 may be occurring in other solid cancerous tissues since hypoxia is often a feature of tumors. Based on our findings, correlative studies would suggest that tumors in which overexpressed CREB and ATF-1 have been identified should present with aberrant levels of 14-3-3ζ. This correlation is in fact present, as determined by expression profiling results available *in silico*. In fact, in the majority of microarray profiles examined, positive correlations exist between *YWHAZ* and CREB family members [22]. An additional member of the 14-3-3 family, 14-3-3ε, also shows a similar profile in the database. This particular isoform harbors a putative CRE element within the promoter of its gene, *YWHAE*. The function of this element has not been confirmed; however, it is possible that CREB family members may control the expression of a sub-set of 14-3-3 family members.

Interestingly, we find that ATF-1 knockdown not only reduces the expression of variant 1c but also caused a marked decrease in variant 1e levels (Figure 7). The endogenous level of variant 1e is relatively low and it is the only variant that repressed translation of 14-3-3ζ in all the cells examined (Figure 3). In the endogenous setting, it is possible that induction of variant 1e by ATF-1 may balance the opposing effect of inducing expression of variant 1c, which promotes translation. This balance can easily be shifted if a mutation were to arise that abolished the binding of ATF-1 to variant 1e. It would be of interest in future studies to identify mutations in variant 1e that prevent ATF-1 from binding that are perhaps correlated with increased tumorigenicity.

With the finding that both CREB and ATF-1 are upregulated in multiple cancers, it seems likely that TNF-α stimulation of these transcription factors may be one mechanism by which cancerous cells can evade TNF-α-induced apoptosis. Indeed, we observed induced binding of ATF-1 and CREB to *YWHAZ* in the presence of TNF-α. Our results suggest that CREB and/or ATF-1 may act to induce activation of this pro-survival gene leading to a feedback loop to reduce TNF-α-induced apoptosis. Thus, a CREB family member inhibitor may be expected to sensitize cells with elevated 14-3-3ζ to TNF-α treatment.

## Materials and Methods

### Cell lines

HeLa and HEK-293 cells were grown in DMEM media and PC3, DU145, LNCap, COLO205, MCF7, A549 and H358 cells were grown in RPMI media. All cell lines used were obtained from ATCC. Cell culture medium was supplemented with 10% FBS, 100 units/ml of penicillin and 100 µg/ml of streptomycin. BEAS cells were maintained in keratinocyte media supplemented with 20 µg/ml bovine pituitary extract and 0.1 ng/ml of human recombinant epidermal growth factor. All cell lines were grown at 37°C in 5% CO_2_ and passed every 3–4 days.

### RNA isolation and RNA-ligase mediated RACE (RLM-RACE)

Total RNA was isolated from cells grown to approximately 80% confluency in 100 mm culture dishes using Qiagens RNeasy kit as described by the manufacturer. Total RNA from HeLa or MCF7 cells was used in the FirstChoice RLM-RACE kit as recommended by the supplier (Invitrogen). Adapter-ligated cDNA products were amplified using a forward primer complementary to the 5′-RACE adapter containing a BamH I restriction site and a reverse primer complementary to exon 2 of *YWHAZ* that contained a Spe I restriction site. PCR products were digested with BamH I and Spe I and cloned into the pBluescript vector. Plasmids isolated from individual clones were purified and sequenced with the T1 primer to identify 5′-RACE products.

### First-strand cDNA synthesis and quantitative real-time PCR (qRT-PCR)

Five µg of total RNA were reverse transcribed using the first-strand cDNA synthesis kit (Invitrogen) followed by qRT-PCR using the BioRad iQ5. Power SYBR green (Applied Biosystems) was used for amplification per manufacturers instructions. Briefly, 2 µl of cDNA were added to a 20 µl reaction containing 1× Power SYBR green master mix and transcript specific primers (refer to Table S1 for primers used). Cycles were conducted with fluorescence intensity measurements acquired following the extension step after each round of amplification.

### Cloning and generation of luciferase constructs

Constructs used to analyze the various 5′-UTRs were generated as follows: Oligos containing the T7 promoter sequences with an EcoR I restriction enzyme site and a HindIII overhang were annealed together and cloned into the HindIII site of pGL3 (Promega). The resulted vector was named T7-pGL3. The various 5′-UTR sequences were amplified from HeLa cell cDNA and were introduced into the T7-PGL3 vector by EcoR I and Nco I sites. All of the constructs were confirmed by sequencing. The primer pairs used to amplify the specific 5′-UTRs are listed in Table S1.

The constructs used for *YWHAZ* 1c promoter studies were generated by cloning the individual sequences upstream of the luciferase gene in pGL4.10 (Promega). The proximal 1c promoter was cut from a previously generated *YWHAZ* 1c luciferase construct with Sac II treated with Klenow, and ligated into EcoR V digested pGL4.10. This new construct contains 806 bp of genomic sequence upstream of the putative transcriptional start site for exon 1c. To generate deletion mutants, the new construct was digested with Sac I and Sma I and the resulted promoter sequence was cloned back into the parent pGL4.10 vector to generate a construct containing 593 bp of genomic sequence upstream of exon 1c. The deletion mutant containing 72 bp of genomic sequence upstream of the putative transcriptional start site of the *YWHAZ* transcript variant 1c was generated by digestion of the -806 vector with Sac I and Aat II. The purified fragment was cloned into pGL4.10. The inserts for the remaining constructs were generated by PCR amplification and cloned into pGL4.10. All forward and reverse primers are listed in Table S1. All constructs were confirmed correct by sequencing.

Luciferase constructs with CRE mutations were generated using QuickChange Site Directed Mutagenesis Kit (Stratagene) according to the manufacturer's protocol. Primers are listed in Table S1.

### Luciferase assays

HeLa cells were seeded in 12-well plates at 1×10^5^ cells/well. The following day, cells were transfected with 150 ng of constructs expressing firefly luciferase, and 20 ng of control vector expressing renilla luciferase using Fugene-HD (Roche). Twenty-four hours after transfection, cells were lysed with 100 µl of passive lysis buffer (Promega). Ten µl of lysate were analyzed for luciferase activity using the Dual Luciferase Kit (Promega) according to manufacturer's instructions. The firefly luciferase activities were normalized against the renilla luciferase activity.

### Nuclear preparation

Nuclear extracts were prepared essentially as described [42]. Protein concentrations were determined by Bio-Rad Protein Assay and nuclear extracts were stored at −80°C.

### Heparin purification

Crude nuclear preparations from HeLa cells (20 mg) were loaded onto a Heparin-agarose column. The column was washed with 10 volumes of loading buffer (20 mM HEPES, pH7.9, 20% glycerol, 0.2 mM EDTA, 0.2 mM EGTA, 2 mM DTT, 0.1 mM PMSF, 50 mM NaCl). Elutions were performed over a 1 M gradient of NaCl and collected in 0.5 ml fractions. Three µl of each of the fractions were subject to binding activity assays by EMSA.

### Electrophoretic mobility shift assays (EMSAs)

Sense and anti-sense oligos for EMSA are listed in Table S1. For the generation of radioactive probes sense and anti-sense oligos labeled with ^32^P were combined at equal molarity, heated to 94°C and slowly cooled to room temperature to allow for annealing. Double stranded probes were separated from single stranded oligos on PAGE gels and subsequently purified. Purified probes were used in combination with nuclear extract to assess binding activities. Briefly, 10 µg of purified extract were added to each well of a 96-well plate containing 30,000 cpm of probe in binding buffer. Plates were incubated with gentle shaking at room-temperature for one hour to induce binding. For supershift assays, 5 µg of each antibody were added at the beginning of the incubation. The following antibodies were used: anti-Atf-1 (#sc-243), anti-Atf-1 (#sc-270), anti-Atf-1 (#sc-H60), anti-Prep-1 (#sc-6245) and anti-Creb (#9197, Cell Signaling). Seventeen µl of each reaction were resolved on 5% PAGE gels. Gels were fixed, dried and exposed to phosphoimaging screens overnight followed by scanning on the Storm (Molecular Dynamics) for further analysis using Image Quant.

### Streptavidin agarose pull down assays

Biotinylated oligos containing the *YWHAZ* CRE region were synthesized and annealed together (refer to Table S1). Oligos, streptavidin-conjugated beads, and 400 µg of nuclear extract were incubated in 1× phosphate buffered saline with protease inhibitors (PBSI) overnight at 4°C for binding. The following day, complexes were precipitated, washed with PBSI three times, and resolved on 12% SDS-PAGE gels. Separated proteins were transferred to nitrocellulose membranes and probed with antisera against CREB and ATF-1 (sc-270) or PREP-1 (sc-6245) to determine protein binding to each of the oligos.

### Chromatin Immunoprecipitation (ChIP)

ChIP experiments were performed as described previously [43]. Briefly, crosslinked DNA-protein complexes were purified from treated HeLa cells and sheared by sonicated. Following extensive washes, precipitates were extracted and the degree of sonication determined by gel electrophoresis. Immunoprecipitation was performed overnight using 5 µg of antibody. The following antibodies were used: anti-Atf-1 (#sc-243), anti-Creb (#9197), and anti-NF-κB-p65 (#sc-372). DNA-protein complexes were recovered with protein A beads and the crosslink reversed. Precipitated DNA was recovered with phenol-cholorform isoamyl alcohol extraction and ethanol precipitation. Pelleted DNA was resuspended in 30 µl of water and 3 µl were used in subsequent real-time PCR amplification. The amount of immunoprecipitated DNA was normalized to 1/30 of the input chromatin. Primer sequences for amplifying *I-κB* and *YWHAZ* promoter regions are listed in Table S1.

### Western blotting

Cells were lysed in 1%-NP-40 lysis buffer (1% NP-40, 10 mM HEPES, pH 7.45, 150 mM NaCl, 10 mg/L aprotin, 10 mg/L leupeptin, 1 mM PMSF). Lysates were clarified by centrifugation, and resolved on 12% SDS-PAGE gels. Proteins were transferred to nitrocellulose membranes and incubated with antisera as indicated in figure legends.

### Short hairpin RNA (shRNA) knockdown and GFP analysis

shRNA constructs were obtained from Open Biosystems. HeLa cells were seeded in 6-well plates at 2×10^5^ cells/well. The following day, 2 µg of each construct were transfected. Twenty-four hours later transfected cells were selected in 1 µg/ml of puromycin. Four days following the initial transfection, total RNA or protein were extracted for analysis of gene-specific knockdown.

### Sequence alignment

Sequence alignment of the *YWHAZ* locus in human, rhesus, mouse, rat, dog, horse, armadillo, elephant, opossum, platypus and tetraodon was performed using the UCSC genome browser (http://genome.ucsc.edu/) [22], [44], [45].

## Supporting Information

Figure S1 **Confirmation that luciferase activity is transcript level independent.**
(PDF)

Table S1 **Oligos used in study design.**
(DOCX)
