# Supplementary material for: Transcriptional Regulation of YWHAZ, the Gene Encoding 14-3-3ζ
Source: PLoS One. 2014 Apr 1;9(4):e93480. doi: 10.1371/journal.pone.0093480 (PMC3972145; doi:10.1371/journal.pone.0093480)
Supplement: Figure S1 — Confirmation that luciferase activity is transcript level independent. (PDF) [file pone.0093480.s001.pdf]

Supplemental Figure 1

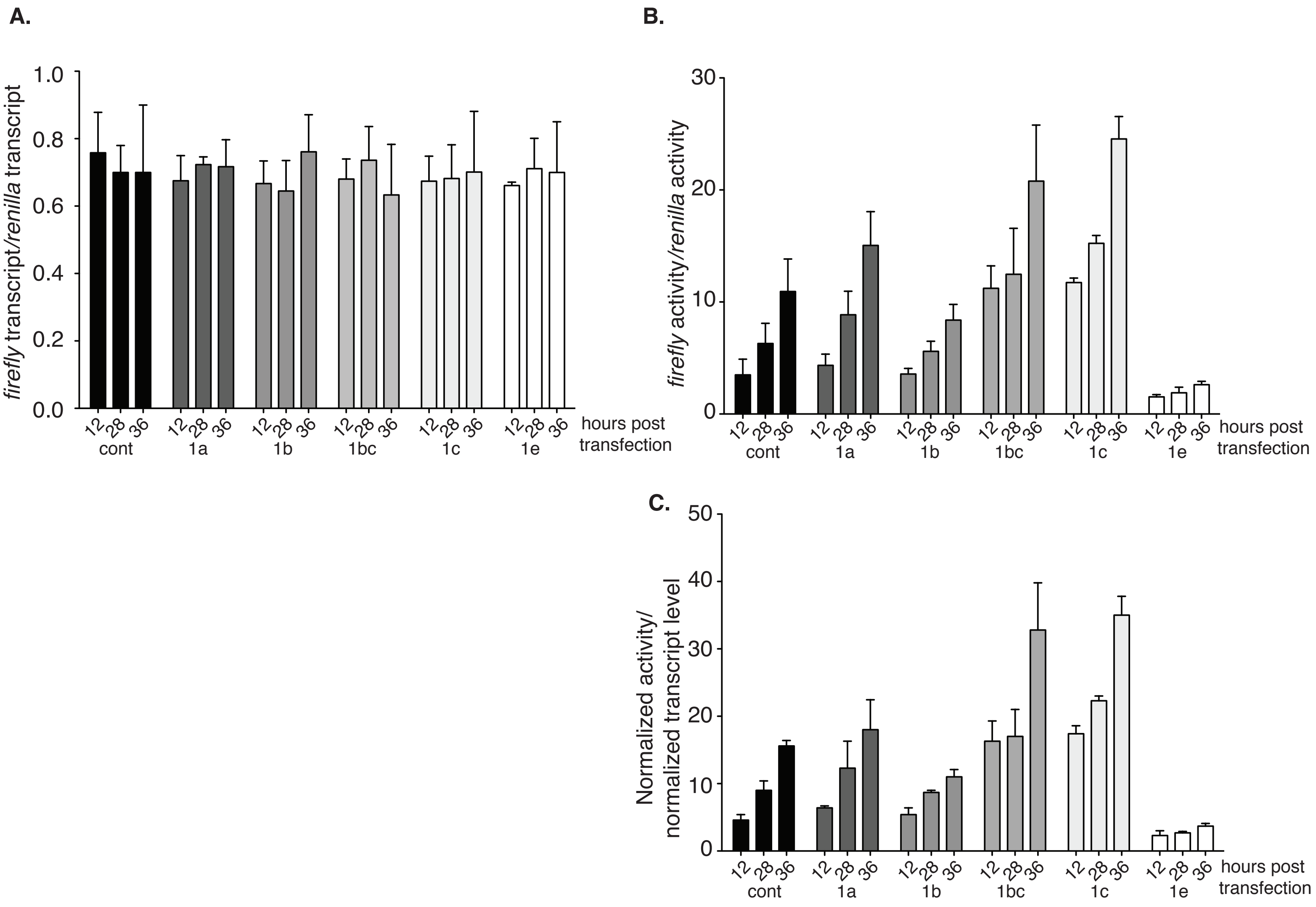

Supplemental Figure 1: Confirmation that luciferase activity is transcript level independent. HeLa cells were transfected with luciferase reporter vectors containing each of the 5′-UTR's and *renialla* control plasmid to normalize transcript levels and luciferase activity. Cells were lysed for protein and total RNA at 12, 28 and 36 hours post transfection. **(A)** qRT-PCR for *firefly* and *renilla* was performed on cDNA obtained from total RNA. *Firefly* transcript levels are graphed relative to *renilla*. **(B)** Isolated protein was assayed for luciferase activity. Data are graphed as *firefly* activity relative to *renilla* activity and are normalized to levels at 12 hours. **(C)** Data from (A) and (B) were combined and graphed as the normalized luciferase activity relative to the normalized transcript levels. Data are shown from one of three independent biological replicates. Error bars for A, B, and C represent standard deviations obtained from four technical replicates.
