# Supplementary material for: Transcriptional Regulation of YWHAZ, the Gene Encoding 14-3-3ζ
Source: PLoS One. 2014 Apr 1;9(4):e93480. doi: 10.1371/journal.pone.0093480 (PMC3972145; doi:10.1371/journal.pone.0093480)
Supplement: Table S1 — Oligos used in study design. (DOCX) [file pone.0093480.s002.docx]

**Supplementary Table 1**

| **Generation of Variant-Specific Luciferase Reporters** | |
| --- | --- |
| 1a forward | 5′-AGAGGAATTCTTTCTCCTTCCCCTTCTTCCGGGCT-3′ |
| 1a reverse | 5′-CTCTCCATGG**GACTGGATGTTCT**TTTTATCTCCTAGAAGC-3′ |
| 1b forward | 5′-AGAGGAATTCGTCCCCTCGCGCAGTCACCGAG-3′ |
| 1b reverse | 5′-CTCTCCATGG**GACTGGATGTTCT**GCTGGCTCGG-3′ |
| 1c forward | 5′-AGAGGAATTCGTTTGACGTCATCGTGCGTGTGGTGC-3′ |
| 1c reverse | 5′-CTCTCCATGG**GACTGGATGTTCT**GTGTCCGGAGTG-3′ |
| 1e forward | 5′-AGAGGAATTCGACACAGATCCGCCATGACAAAGGAGGAGA-3′ |
| 1e reverse | 5′-CTCTCCATGG**GACTGGATGTTCT**GACTTGAGACGTC-3′ |
| Underlined nucleotides in forward and reveres primers indicate EcoRI and NcoI sites respectively. Bold nucleotides are shared between all variants. These are the nucleotides preceding the start methionine in exon 2. | |
| **Generation of Variant 1c Truncation Luciferase Reporters** | |
| -507 forward | 5′-AAGAGCTCTCCCGGATGTTGCTGAACGGGA-3′ |
| -453 forward | 5′-AAGAGCTCCAGACGGGCTGAGCGAAAGCTAAG-3′ |
| -376 forward | 5′-AAGAGCTCCAGAGGCTGCAGGGGAGAGGGA-3′ |
| -284 forward | 5′-AAGAGCTCAAGCAGCCTCCTGCCCTAGGTGC-3′ |
| -166 forward | 5′-AAGAGCTCCGGGCGGGCAGGACGAGCGCCGGG-3′ |
| -85 forward | 5′-AAGAGCTCCAGCGTTTGACGTCATCGTGCGTGTGGTG-3′ |
| +1 forward | 5′-AAGAGCTCGCCTGTGAGCAGCGAGATCCA-3′ |
| reverse | 5′-CTCTGCTAGCGACTGGATGTTCTGTGTCCGGAGTG-3′ |
| Underlined nucleotides in forward and reveres primers indicate SacI and NheI sites respectively | |
| **Generation of CRE-Mutant Luciferase Reporters** | |
| CRE Mutag. for | 5′-GCCATAGCAGCGTTTGAGATCATCGTGCGTGTGG-3′ |
| CRE Mutag. rev | 5′-CCACACGCACGATGATCTCAAACGCTGCTATGGC-3′ |
| Underlined nucleotides indicates location of two nucleotide mutation | |
| **RT-PCR Primer Pairs** | |
| 1a forward | 5′-GGCTCCCGTCCCGGCTC-3′ |
| 1b forward | 5′-AGATCCCGCGCTCTCGATTGG-3′ |
| 1c forward | 5′-GTGAGCAGCGAGATCCAGGG-3′ |
| exon 2 forward | 5′-AGCAGGCTGAGCGATATGAT-3′ |
| Ex3-4 Rev | 5′-CGACAATCCCTTTCTTGTC-3′ |
| **EMSA/SAPD Probes** | |
| EMSA wild-type | 5′-TAGCAGCGTTTGACGTCATCGTGCGTGT-3′ (and complement) |
| EMSA mutant | 5′-TAGCAGCGTTTGAGATCATCGTGCGTGT-3′ (and complement) |
| Underlined nucleotides indicates location of two nucleotide mutation | |
| **ChIP Primer Pairs** | |
| I-κB | 5′-GACGACCCCAATTCAAATCG-3′ |
|  | 5′-TCAGGCTCGGGGAATTTCC-3′ |
| *YWHAZ* | 5′-GAAGCCGAGCGGGGTGGGAGGAGT-3′ |
|  | 5′-GGCGGCGGACGGACGGGCTCAG-3′ |
